# Clinicopathological characteristics and survival outcomes in patients with synchronous lung metastases upon initial metastatic breast cancer diagnosis in Han population

**DOI:** 10.1186/s12885-021-09038-2

**Published:** 2021-12-14

**Authors:** Shaoyan Lin, Hongnan Mo, Yiqun Li, Xiuwen Guan, Yimeng Chen, Zijing Wang, Binghe Xu

**Affiliations:** Department of Medical Oncology, National Cancer Center/National Clinical Research Center for Cancer/Cancer Hospital, Chinese Academy of Medical Sciences & Peking Union Medical College, No.17, Panjiayuan Nanli, Chaoyang District, Beijing, 100021 China

**Keywords:** Breast neoplasms, Lung, Neoplasm metastasis, Prognosis, Survival

## Abstract

**Background:**

We investigated the clinicopathological characteristics and survival of breast cancer lung metastases (BCLM) patients at initial diagnosis of metastatic breast cancer (MBC) in the Han population.

**Methods:**

We attained clinical data of 3155 MBC patients initially diagnosed between April 2000 and September 2019 from the China National Cancer Center and finally included 2263 MBC patients in this study, among which 809 patients presented with lung metastases at first MBC diagnosis. The risk factors for BCLM were determined using multivariate logistic regression analysis and the prognostic factors of BCLM patients were assessed by univariate and multivariate Cox regression analyses.

**Results:**

Patients with triple-negative subtype (42.3%) harbored the highest incidence proportions of lung metastases. Age ≥ 50 years, Eastern Cooperative Oncology Group (ECOG) 2, M1, hormone receptor-negative (HR-)/human epidermal growth factor receptor 2-positive (HER2) + subtype, triple-negative subtype and disease-free survival (DFS) > 2 years were remarkably associated with higher incidence of lung metastases, while invasive lobular carcinoma (ILC) and bone metastases were significantly correlated with lower odds of lung metastases at diagnosis. The median survival of BCLM patients was 41.7 months, with triple-negative subtype experiencing the worst prognosis of 26.8 months. ECOG 2, triple-negative subtype, liver metastases, multi-metastatic sites and DFS ≤ 2 years were significantly correlated with poor survival of BCLM patients.

**Conclusions:**

Our study provides essential information on clinicopathological features and survival outcomes of BCLM patients at initial diagnosis of MBC in China.

## Background

Lung metastasis is the second most frequent distant metastases of breast cancer [1, 2], clinically presenting in 15–25% of metastatic breast cancer (MBC) patients [3, 4]. Autopsy data of 197 women dying with MBC over a period of 50 years revealed that 80.7% of patients had lung or pleura metastases [5]. A population-based study indicated that the median survival of 3372 patients with lung metastases at primary breast cancer diagnosis was 21 months [6]. Although the prognosis of MBC patients with metastases confined to lungs is not so poor as brains or livers [7], most patients are considered incurable and the treatment is still intractable. With an occult onset, lung metastases from breast cancer usually present asymptomatically and progress aggressively without appropriate care [8]. Systemic treatments including chemotherapy, targeted therapy and hormone therapy are recommended for patients with breast cancer lung metastases (BCLM) [9] and pulmonary metastasectomy is considerable for properly selected cases [10]. The early detection of lung metastasis and the precise estimation of outcome may benefit breast cancer patients in clinical practice, thus achieving long-term survival. However, the clinicopathological characteristics and the risk factors that affect the incidence and prognosis of BCLM remain poorly identified in the Han population.

In this article, we summarized the clinicopathological features and explored the risk factors associated with the morbidity and mortality of BCLM in newly diagnosed MBC patients in China, which may help identify cases with higher odds of lung metastases and worse survival. Early intervention and multidisciplinary treatment for BCLM patients are of utmost importance.

## Methods

This work was approved by the institutional review board of National Cancer Center/National Clinical Research Center for Cancer/Cancer Hospital, Chinese Academy of Medical Sciences and Peking Union Medical College. All methods were carried out in accordance with relevant guidelines and regulations. The study methods referred to the previous report [11].

### Study population

We attained clinical data of 3155 MBC patients initially diagnosed between April 2000 and September 2019 from the China National Cancer Center database. The database was generated and maintained by medical staff, drawn from the medical records in the hospital information system of China National Cancer Center. Several studies based on this database have been published [11–13]. We removed patients with unknown tumor receptor status (*n* = 579), unknown distant metastases (*n* = 65) and follow-up no more than 1 month since the initial diagnosis of MBC (*n* = 254) from this cohort, finally leaving 2263 patients for incidence analysis. Among these, 809 cases presented with lung metastases (including lymphangitic carcinomatosis and pleural disease) upon initial MBC diagnosis. Lung metastases were identified by enhanced chest CT scan and 220/809 (27.2%) patients were biopsy proven. Based on the guidelines in our center, lung biopsy was not essential unless the imaging was uncertain. With the improvement of the guidelines, lung biopsy was also considerable for the sake of therapy guidance or patient wishes. Telephone calls or clinical visits were used to follow up patients further to June 30, 2019 or date of their deaths.

### Study variables

Study variables, including age at initial MBC diagnosis, Eastern Cooperative Oncology Group (ECOG) grade, pathological type, TNM stage of primary breast cancer, tumor receptor status, number and type of metastatic sites, disease-free survival (DFS) between primary breast cancer diagnosis and metastatic recurrence, first-line therapy and overall survival (OS) from the onset of metastasis to death were retrospectively collected. DFS was divided as ≤2 years, > 2 years and patients with de-novo diseases were classified as M1 group. Cancers with 1–100% estrogen receptor or progesterone receptor routine immunohistochemistry (IHC) staining were considered hormone receptor-positive (HR+). Human epidermal growth factor receptor 2 (HER2) overexpression was defined as IHC3+ or in the case of IHC2+, fluorescent in-situ hybridization (FISH) positive. The HER2 status was determined according to the American Society of Clinical Oncology (ASCO)/College of American Pathologists (CAP) guidelines. Since the ASCO/CAP guidelines have updated across years, the HER2 status was evaluated based on different versions in certain years (2000–2019). The receptor status of metastatic tumors was re-assessed in 512/2263 (22.7%) cases. Breast cancer subtypes were divided as HR+/HER2-, HR−/HER2+, HR+/HER2+ and triple-negative (HR−/HER2-), based on primary tumor. Tumor staging of the primary tumor was based on the 8th American Joint Committee on Cancer (AJCC) TNM staging system.

### Statistical analysis

Chi-square or Fisher’s exact test were used for category variables to compare the clinicopathological features among different subtypes in patients with lung metastases. Incidence of lung metastases was defined as the number of BCLM patients divided by the total number of MBC patients. We performed multivariate logistic regression to explore factors associated with the presence of lung metastases upon initial diagnosis of MBC. We calculated odds ratios (ORs) and 95% confidence intervals (CIs) in the model. Kaplan-Meier method was utilized to estimate the survival within subsets and log-rank test was used to analyze the differences. We conducted univariate and multivariate Cox regression analyses to investigate the independent predictive factors significantly associated with the prognosis of BCLM patients. All the statistics were analyzed using SPSS statistical software version 23.0 package. A two-sided *p* value of 0.05 or less was significantly different.

## Results

### Patient characteristics

A total of 2263 MBC patients were enrolled in final cohort, of which 35.7% (809) synchronously presented with lung metastases upon initial MBC diagnosis and Table 1 listed their clinicathological characteristics stratified by breast cancer subtype. It showed that 15.1% (122) of BCLM patients were diagnosed with de novo metastatic disease (M1). Only 43.9% (108/246) of BCLM patients with HER2-positive received anti-HER2 therapy during first line. BCLM patients with HR+/HER2-, HR−/HER2+, HR+/HER2+ and triple-negative subtypes accounted for 47.7, 14.3, 16.1 and 21.9%, respectively. Compare with other subsets, triple-negative patients with lung metastases were younger (*p =* 0.015), had an earlier N-stage of primary breast cancer (*p =* 0.005) and a shorter DFS (*p* < 0.001), presented with more recurrent diseases (*p =* 0.002) and less liver metastases (*p =* 0.001). HER2+ (HR−/HER2+ and HR+/HER2+) patients with BCLM were more frequently diagnosed with de novo stage IV breast cancer than HER2- (HR+/HER2- and triple-negative) patients (*p =* 0.002). BCLM patients with HR+/HER2- subtype had the highest rate of bone metastases (*p* < 0.001).

**Table 1:** Clinicopathological characteristics of patients with lung metastases upon initial metastatic breast cancer diagnosis according to breast cancer subtype

Characteristic	HR+/HER2-, N (%)	HR−/HER2+, N (%)	HR+/HER2+, N (%)	Triple-negative, N (%)	*p* value
All patients	386 (47.7)	116 (14.3)	130 (16.1)	177 (21.9)	
Age					0.015
< 50	172 (44.6)	44 (37.9)	62 (47.7)	99 (55.9)	
≥ 50	214 (55.4)	72 (62.1)	68 (52.3)	78 (44.1)	
ECOG					0.194
0	91 (23.6)	24 (20.7)	23 (17.7)	46 (26.0)	
1	278 (72.0)	88 (75.9)	102 (78.5)	117 (66.1)	
2	17 (4.4)	4 (3.4)	5 (3.8)	14 (7.9)	
Pathological type					0.552
IDC	355 (92.0)	111 (95.7)	125 (96.2)	168 (94.9)	
ILC	9 (2.3)	1 (0.9)	2 (1.5)	3 (1.7)	
Others	22 (5.7)	4 (3.4)	3 (2.3)	6 (3.4)	
T-stage					0.065
T1	104 (26.9)	24 (20.7)	32 (24.6)	47 (26.6)	
T2	169 (43.8)	50 (43.1)	52 (40.0)	83 (46.9)	
T3	20 (5.2)	6 (5.2)	5 (3.8)	17 (9.6)	
T4	18 (4.7)	12 (10.3)	9 (6.9)	6 (3.4)	
Unknown	75 (19.4)	24 (20.7)	32 (24.6)	24 (13.6)	
N-stage					0.005
N0	113 (29.3)	25 (21.6)	30 (23.1)	65 (36.7)	
N1	96 (24.9)	23 (19.8)	43 (33.1)	45 (25.4)	
N2	66 (17.1)	22 (19.0)	23 (17.7)	31 (17.5)	
N3	67 (17.4)	35 (30.2)	20 (15.4)	19 (10.7)	
Unknown	44 (11.4)	11 (9.5)	14 (10.8)	17 (9.6)	
M-stage					0.002
M0	339 (87.8)	88 (75.9)	103 (79.2)	157 (88.7)	
M1	47 (12.2)	28 (24.1)	27 (20.8)	20 (11.3)	
Liver metastases					0.001
No	308 (79.8)	85 (73.3)	88 (67.7)	152 (85.9)	
Yes	78 (20.2)	31 (26.7)	42 (32.3)	25 (14.1)	
Brain metastases					0.625
No	370 (95.9)	108 (93.1)	124 (95.4)	170 (96.0)	
Yes	16 (4.1)	8 (6.9)	6 (4.6)	7 (4.0)	
Bone metastases					< 0.001
No	226 (58.5)	91 (78.4)	89 (68.5)	134 (75.7)	
Yes	160 (41.5)	25 (21.6)	41 (31.5)	43 (24.3)	
Number of metastatic sites					0.001
1	104 (26.9)	28 (24.1)	42 (32.3)	53 (29.9)	
2	108 (28.0)	48 (41.4)	37 (28.5)	74 (41.8)	
≥ 3	174 (45.1)	40 (34.5)	51 (39.2)	50 (28.2)	
Anti-HER2 therapy during first line					0.429
Yes	–	54 (46.6)	54 (41.5)		
No	–	62 (53.4)	76 (58.5)		
DFS					< 0.001
≤ 2 years	83 (21.5)	47 (40.5)	36 (27.7)	94 (53.1)	
> 2 years	256 (66.3)	41 (35.3)	67 (51.5)	63 (35.6)	
M1	47 (12.2)	28 (24.2)	27 (20.8)	20 (11.3)	

*HR* hormone receptor, *HER2* human epidermal growth factor receptor 2, *ECOG* Eastern Cooperative Oncology Group, *IDC* invasive ductal carcinoma, *ILC* invasive lobular carcinoma, *DFS* disease-free survival

Table 2 displayed the incidence of patients with lung metastases stratified by breast cancer subtype. HR+/HER2-, HR−/HER2+, HR+/HER2+ and triple-negative subtypes accounted for 52.1, 13.3, 16.1 and 18.5% of the entire MBC population, respectively. Patients with triple-negative subtype (42.3%) harbored the highest incidence proportions of lung metastases.

**Table 2:** Incidence of patients with lung metastases at first metastatic breast cancer diagnosis stratified by breast cancer subtype

	All metastatic patients, N (%)	With lung metastases	Incidence of lung metastases, %
HR+/HER2-	1180 (52.1)	386	32.7
HR−/HER2+	300 (13.3)	116	38.6
HR+/HER2+	365 (16.1)	130	35.6
Triple-negative	418 (18.5)	177	42.3
All subtypes	2263 (100.0)	809	35.7

*HR* hormone receptor, *HER2* human epidermal growth factor receptor 2

Association between the presence of lung metastases at initial MBC diagnosis and variables assessed by multivariate logistic regression was showed in Table 3. Age ≥ 50 years (vs. < 50 years, OR = 1.29, 95% CI = 1.08–1.54, *p =* 0.005), ECOG 2 (vs. ECOG 0, OR = 1.67, 95% CI = 1.04–2.67, *p =* 0.033), M1 (vs. M0, OR = 1.42, 95% CI =1.05–1.92, *p =* 0.022), HR−/HER2+ subtype (vs. HR+/HER2-, OR = 1.40, 95% CI = 1.06–1.85, *p =* 0.020), triple-negative subtype (vs. HR+/HER2-, OR = 1.63, 95% CI = 1.28–2.09, *p* < 0.001) and DFS > 2 years (vs. DFS ≤ 2 years, OR = 1.74, 95% CI = 1.42–2.14, *p* < 0.001) were remarkably associated with higher incidence of lung metastases at diagnosis. Invasive lobular carcinoma (ILC) (vs. invasive ductal carcinoma (IDC), OR = 0.39, 95% CI = 0.22–0.70, *p =* 0.002) and bone metastases (vs. without bone metastases, OR = 0.74, 95% CI = 0.61–0.90, *p =* 0.002) were significantly correlated with lower odds of lung metastases at diagnosis.

**Table 3:** Multivariate logistic regression for the presence of lung metastases at initial diagnosis of metastatic breast cancer

Characteristic	OR (95% CI)	*p* value
Age		
< 50	Reference	
≥ 50	1.29 (1.08, 1.54)	0.005
ECOG		
0	Reference	
1	1.16 (0.94, 1.43)	0.162
2	1.67 (1.04, 2.67)	0.033
Pathological type		
IDC	Reference	
ILC	0.39 (0.22, 0.70)	0.002
Others	1.36 (0.81, 2.28)	0.241
T-stage		
T1	Reference	
T2	1.07 (0.86, 1.35)	0.536
T3	0.77 (0.52, 1.14)	0.190
T4	1.33 (0.84, 2.11)	0.223
Unknown	0.84 (0.63, 1.12)	0.228
N-stage		
N0	Reference	
N1	1.03 (0.81, 1.32)	0.787
N2	0.99 (0.75, 1.30)	0.927
N3	0.85 (0.64, 1.13)	0.262
Unknown	1.08 (0.75, 1.56)	0.672
M-stage		
M0	Reference	
M1	1.42 (1.05, 1.92)	0.022
Subtype		
HR+/HER2-	Reference	
HR−/HER2+	1.40 (1.06, 1.85)	0.020
HR+/HER2+	1.19 (0.92, 1.53)	0.188
Triple-negative	1.63 (1.28, 2.09)	< 0.001
Liver metastases		
No	Reference	
Yes	0.82 (0.66, 1.01)	0.067
Brain metastases		
No	Reference	
Yes	1.12 (0.72, 1.74)	0.608
Bone metastases		
No	Reference	
Yes	0.74 (0.61, 0.90)	0.002
DFS		
≤ 2 years	Reference	
> 2 years	1.74 (1.42, 2.14)	< 0.001
M1	1.42 (1.05, 1.92)	0.022

*OR* odds ratio, *CI* confidence interval, *ECOG* Eastern Cooperative Oncology Group, *IDC* invasive ductal carcinoma, *ILC* invasive lobular carcinoma, *HR* hormone receptor, *HER2* human epidermal growth factor receptor 2, *DFS* disease-free survival

### Survival

**Fig. 1:**
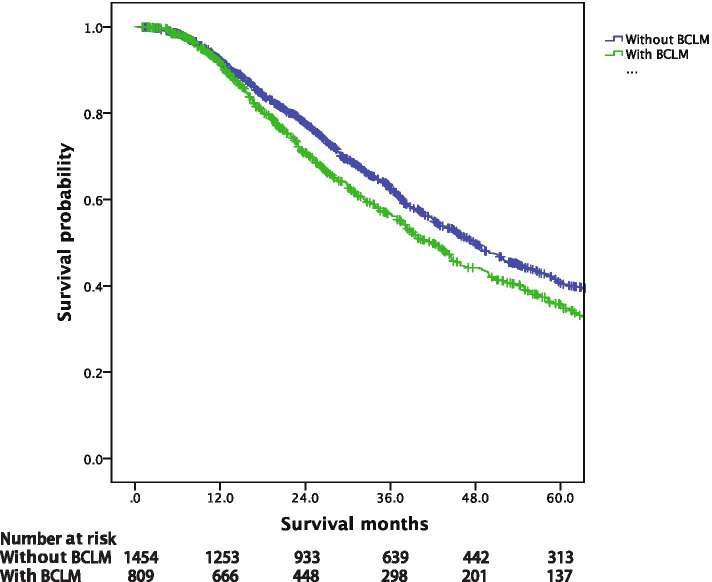
Overall survival of metastatic breast cancer patients with or without BCLM. BCLM, breast cancer lung metastases

**Fig. 2:**
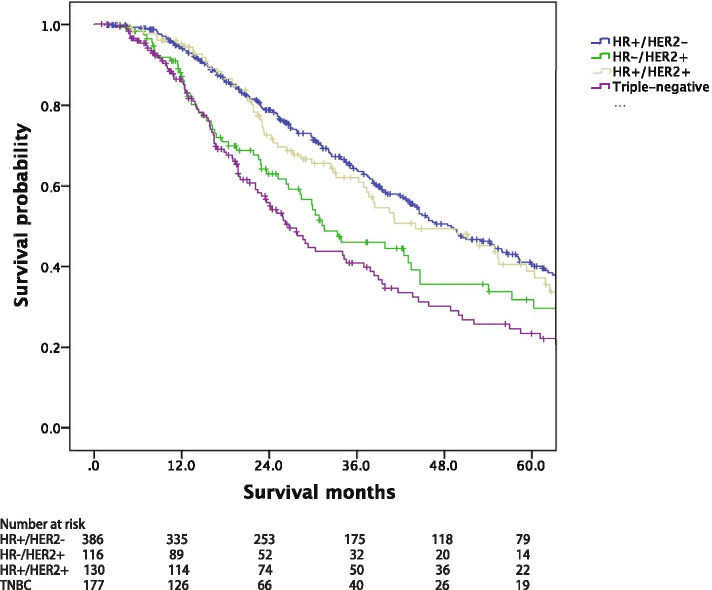
Overall survival of BCLM patients according to breast cancer subtype. BCLM, breast cancer lung metastases, HR, hormone receptor, HER2, human epidermal growth factor receptor 2, TNBC, triple-negative

The median survival among the whole MBC cohort was 45.4 months, with a median follow-up of 61.6 months. Figure 1 showed that the prognosis of patients with lung metastases upon MBC diagnosis (median OS, 41.7 months) was significantly worse than those without lung metastases (median OS, 47.9 months, *p =* 0.001). Figure 2 provided the survival of BCLM patients according to breast cancer subtype. The survival of BCLM patients with HR+/HER2- subtype (49.0 months) was the longest, while triple-negative (26.8 months, *p* < 0.001) the shortest. BCLM patients with HR−/HER2+ (vs. HR+/HER2-, *p =* 0.009) and HR+/HER2+ (vs. HR+/HER2-, *p =* 0.746) subtypes experienced the median OS of 31.6 and 44.1 months, respectively.

The prognostic factors of BCLM patients assessed by univariate and multivariate Cox regression analyses were presented in Table 4. The significant variables with *p* value < 0.05 in univariate analysis were further included in multivariate Cox regression model. ECOG 2 (vs. ECOG 0, HR = 1.75, 95% CI = 1.12–2.73, *p =* 0.015), triple-negative subtype (vs. HR+/HER2-, HR = 1.76, 95% CI = 1.36–2.29, *p* < 0.001), liver metastases (vs. without liver metastases, HR = 2.19, 95% CI = 1.70–2.82, *p* < 0.001), 2 metastatic sites (vs. 1 metastatic site, HR = 1.76, 95% CI = 1.34–2.31, *p* < 0.001), and ≥ 3 metastatic sites (vs. 1 metastatic site, HR = 1.74, 95% CI = 1.24–2.44, *p* = 0.001) were significantly correlated with poor survival of BCLM patients. DFS > 2 years (vs. DFS ≤ 2 years, HR = 0.66, 95% CI = 0.53–0.83, *p* < 0.001) predicted favorable prognosis of BCLM patients.

**Table 4:** Univariate and multivariate cox regression analyses of OS in BCLM patients

Univariable analysis			Multivariable analysis		
Characteristic	Hazard ratio (95% CI)	*p* value	Characteristic	Hazard ratio (95% CI)	*p* value
Age			Age		
< 50	Reference		< 50		
≥50	1.04 (0.86, 1.25)	0.715	≥50		
ECOG			ECOG		
0	Reference		0	Reference	
1	1.23 (0.96, 1.59)	0.099	1	1.13 (0.87, 1.46)	0.351
2	2.62 (1.71, 4.01)	< 0.001	2	1.75 (1.12, 2.73)	0.015
Pathological type					
IDC	Reference				
ILC	0.97 (0.53, 1.76)	0.912			
Others	0.79 (0.49, 1.27)	0.324			
T-stage			T-stage		
T1	Reference		T1	Reference	
T2	1.17 (0.92, 1.47)	0.197	T2	1.05 (0.83, 1.34)	0.682
T3	1.51 (1.01, 2.26)	0.044	T3	1.10 (0.72, 1.69)	0.654
T4	1.41 (0.94, 2.11)	0.095	T4	1.11 (0.70, 1.76)	0.654
Unknown	0.93 (0.70, 1.24)	0.625	Unknown	0.98 (0.70, 1.37)	0.896
N-stage			N-stage		
N0	Reference		N0	Reference	
N1	1.17 (0.90, 1.52)	0.241	N1	1.05 (0.80, 1.38)	0.719
N2	1.34 (1.01, 1.77)	0.045	N2	1.06 (0.78, 1.44)	0.708
N3	1.79 (1.36, 2.36)	< 0.001	N3	1.26 (0.91, 1.74)	0.165
Unknown	1.19 (0.84, 1.69)	0.330	Unknown	1.01 (0.65, 1.55)	0.977
M-stage					
M0	Reference				
M1	1.15 (0.89, 1.49)	0.296			
Subtype			Subtype		
HR+/HER2-	Reference		HR+/HER2-	Reference	
HR−/HER2+	1.43 (1.08, 1.90)	0.013	HR−/HER2+	1.35 (1.00, 1.83)	0.051
HR+/HER2+	1.04 (0.79, 1.37)	0.769	HR+/HER2+	1.04 (0.78, 1.38)	0.788
Triple-negative	1.73 (1.36, 2.19)	< 0.001	Triple-negative	1.76 (1.36, 2.29)	< 0.001
Liver metastases			Liver metastases		
No	Reference		No	Reference	
Yes	2.71 (2.20, 3.35)	< 0.001	Yes	2.19 (1.70, 2.82)	< 0.001
Brain metastases					
No	Reference				
Yes	1.40 (0.94, 2.10)	0.100			
Bone metastases			Bone metastases		
No	Reference		No	Reference	
Yes	1.43 (1.18, 1.74)	< 0.001	Yes	1.13 (0.88, 1.44)	0.344
Number of metastatic sites			Number of metastatic sites		
1	Reference		1	Reference	
2	1.86 (1.44, 2.40)	< 0.001	2	1.76 (1.34, 2.31)	< 0.001
≥3	2.42 (1.89, 3.09)	< 0.001	≥3	1.74 (1.24, 2.44)	0.001
DFS			DFS		
≤2 years	Reference		≤2 years	Reference	
> 2 years	0.59 (0.48, 0.72)	< 0.001	> 2 years	0.66 (0.53, 0.83)	< 0.001
M1	0.81 (0.61, 1.08)	0.147	M1	0.76 (0.55, 1.06)	0.105
First-line therapy			First-line therapy		
Single-agent chemotherapy	Reference		Single-agent chemotherapy	Reference	
Combination therapy	0.71 (0.47, 1.07)	0.099	Combination therapy	0.69 (0.46, 1.05)	0.085
Endocrine therapy	0.43 (0.25, 0.75)	0.003	Endocrine therapy	0.70 (0.39, 1.25)	0.223

*OS* overall survival, *BCLM* breast cancer lung metastases, *CI* confidence interval, *ECOG* Eastern Cooperative Oncology Group, *IDC* invasive ductal carcinoma, *ILC* invasive lobular carcinoma, *HR* hormone receptor, *HER2* human epidermal growth factor receptor 2, *DFS* disease-free survival

## Discussion

In this retrospective study, we described the clinicopathological characteristics and analyzed the prognosis of patients with synchronous lung metastases at initial MBC diagnosis in China. We identified 809 patients with BCLM upon newly diagnosis of MBC, accounting for 35.7% of all MBC patients. Compared with other groups, patients with triple-negative subtype had the highest percentage of lung metastases, consistent with previous findings [14–16]. The incidence of lung metastasis in triple-negative breast cancer (TNBC) could reach up to 40% [17], similar with 42.3% in our data. Additionally, the prognosis of BCLM patients differed remarkably in tumor subtypes, varying between 26.8 months of triple-negative subtype and 49.0 months of HR+/HER2- subtype.

Our study confirmed the results that TNBC was more aggressive and preferred to develop lung metastases. The molecular mechanisms underlying TNBC metastasis to lung might offer therapeutic targets for clinical prevention and management. Minn et al. [18] identified fascin as a mediator promoting basal-like breast cancer metastasis to lung, due to its close association with cell motility. Iriondo et al. [19] observed that inhibition of transforming growth factor-β1-activated kinase-1 (TAK1) could suppress lung metastasis in TNBC, which might provide a novel target for impairing TNBC lung metastasis. A single mutation on microrchidia family CW-type zinc finger 2 (MORC2) promoted TNBC lung metastasis by regulating heterogeneous nuclear ribonucleoprotein M (hnRNPM)- mediated CD44 splicing, which indicated that the knockdown of hnRNPM might reduce lung metastatic potential of TNBC cells with mutant MORC2 [20]. Another research revealed that the overexpression of transcription and export complex 2 subunit (ENY2) could promote TNBC progression and lung metastasis both in vitro and in vivo [21]. Further mechanisms clarifying TNBC lung metastasis are certainly worth exploring, which may provide potential targets for new drugs.

Our data also indicated that patients with older age and worse performance status were more likely to present with lung metastases at initial MBC diagnosis. The increasing risk of lung metastases associated with aging was consistently found in population-based studies [6, 22]. On the contrary, previous studies observed that younger patients had a higher risk of liver metastases [5, 23]. Increased levels of urinary prostaglandin E-metabolite (PGE-M), a biomarker of inflammation, were observed in aging and lung metastases in patients with breast cancer [24]. Levels of multiple proinflammatory mediators, known as inducers of cyclooxygenase-2 (COX-2) and prostaglandin E_2_ (PGE_2_) synthesis, elevated during aging, which contributed to the increase of PGE-M, a catabolic product of PGE_2_ [25]. Overexpression of COX-2 in tumor cells within the lung metastases could explain the increased level of PGE-M [26]. It’s possible that age-related inflammatory conditions mediated breast cancer metastasis to the lung. The predictive features associated with different metastatic sites may help clinicians distinguish patients with distinct organ-specific metastases during the clinical practice.

The BCLM patients in our data achieved a median OS of 41.7 months since MBC diagnosis, among which triple-negative subtype experienced the worst outcome of 26.8 months and HR+/HER2- subtype the best of 49.0 months. The prognosis of MBC patients varied remarkably by the metastatic organs, with the best for bone, followed by lung, liver and the worst for brain metastases [7, 27]. Previous findings recorded a survival ranging from 21.0 to 58.5 months in MBC patients with lung metastases [1, 6, 28]. A pulmonary metastasectomy study reported a median survival of 23.6 months in TNBC patients with an isolated and limited number of lung metastases, significantly poorer than HR+ or HER2+ patients [29]. A population-based research showed that TNBC patients with metastases confined to lung had a median OS of only 14.0 months [30]. TNBC is still lethal and remains intractable to existing treatments, extremely desirable for novel therapies to improve the prognosis.

We also identified prognostic factors for survival of BCLM patients and found that worse performance status, triple-negative subtype, the simultaneous presence of liver metastases, multi-metastatic sites and shorter DFS were significantly correlated with poor outcome. Multiple sites of first metastases had significantly unfavorable prognosis than single site first metastases [31, 32]. In our data, the extrapulmonary metastases had 1.7 times of mortality risk than lung-only metastases at MBC diagnosis. Brain metastases also worsen the outcome of BCLM patients but the difference did not reach significance, probably due to the late onset of brain metastases during the clinical course, with an incidence of only 6.90 to 7.56% in newly MBC diagnosis patients [32–34]. BCLM patients with DFS shorter than 2 years experienced poorer survival, which indicated the intrinsic aggressiveness of the tumors.

There were some limitations in our study. Firstly, discordance in tumor phenotype has been reported in multiple studies [35], but we did not have enough information on the receptor status of metastatic tumors, which might cause some bias in the analysis of incidence and survival outcomes when stratified by breast cancer subtype. Secondly, the fact that less than half of BCLM patients with HER2-positive received anti-HER2 therapy during first line limits the generalizability of the outcome results. Additionally, the number of lung lesions was an important risk factor for BCLM patients [36], but it was not documented in detail in our database. Finally, the retrospective nature of this research and relatively small population require future studies to confirm the results.

## Conclusions

Our study provides essential information on clinicopathological features and survival outcomes of BCLM patients at initial diagnosis of MBC in China. The risk factors identified here help to screen breast cancer patients with high odds of lung metastases and BCLM patients with high risk of mortality. The early detection of metastases and proper evaluation of prognosis in clinical practice are beneficial to optimize the disease outcomes.

## Data Availability

The data used during the current study are available from the corresponding author on reasonable request.

## Abbreviations

MBC: Metastatic breast cancer
BCLM: Breast cancer lung metastases
ECOG: Eastern Cooperative Oncology Group
DFS: Disease-free survival
OS: Overall survival
IHC: Immunohistochemistry
HR: Hormone receptor
HER2: Human epidermal growth factor receptor 2
FISH: Fluorescent in-situ hybridization
ASCO/CAP: American Society of Clinical Oncology/College of American Pathologists
AJCC: American Joint Committee on Cancer
ILC: Invasive lobular carcinoma
IDC: Invasive ductal carcinoma
OR: Odds ratio
CI: Confidence interval
TNBC: Triple-negative breast cancer
TAK1: Transforming growth factor-β1-activated kinase-1
MORC2: Microrchidia family CW-type zinc finger 2
hnRNPM: Heterogeneous nuclear ribonucleoprotein M
ENY2: Transcription and export complex 2 subunit
PGE-M: Prostaglandin E-metabolite
COX-2: Cyclooxygenase-2
PGE_2_: Prostaglandin E_2_
